# Environmental Niche Modelling Predicts a Contraction in the Potential Distribution of Two Boreal Owl Species under Different Climate Scenarios

**DOI:** 10.3390/ani12223226

**Published:** 2022-11-21

**Authors:** Kristina Cerman, Draženko Rajković, Biljana Topić, Goran Topić, Peter Shurulinkov, Tomaž Mihelič, Juan D. Delgado

**Affiliations:** 1 Faculty of Environmental Sciences, Czech University of Life Sciences Prague, Kamýcká 129, Suchdol, 165 00 Prague, Czech Republic; 2 Center for Biodiversity Research, Maksima Gorkog 40/3, 21000 Novi Sad, Serbia; 3 Ornithological Society ‘‘Naše Ptice’’, Semira Frašte 6/14, 71000 Sarajevo, Bosnia and Herzegovina; 4 National Museum of Natural History—Sofia, Bulgarian Academy of Sciences, 1 Tsar Osvoboditel Blvd., 1000 Sofia, Bulgaria; 5 DOPPS-BirdLife Slovenia, Tržaška Cesta 2, 1000 Ljubljana, Slovenia; 6 Department of Physical, Chemical and Natural Systems, Ecology Area, Faculty of Experimental Sciences, Universidad Pablo de Olavide, E-41013 Sevilla, Spain

**Keywords:** *Aegolius funereus*, Balkan Peninsula, climate change, *Glaucidium passerinum*, MaxEnt, species distribution modelling, suitability modelling, refugia

## Abstract

**Simple Summary:**

Studying species distribution modelling in the face of climate change provides more insight into how endangered species are affected by these changes. Therefore, we studied two locally endangered owl species, the Boreal and Eurasian Pygmy Owl, in the Balkan Peninsula to better understand their current and future distribution. We aimed to perform species distribution modelling for these two targeted owl species in current climate and future predicted climate scenarios. We quantified highly suitable areas for both species currently and in future climate scenarios. Additionally, we looked at the size of the areas of future species’ refugia where environmental factors might be suitable for the species. Results showed that the future highly suitable area for Boreal Owl shrunk compared to the current area in all climate scenarios; however, for Eurasian Pygmy Owl, the results did not follow such a clear trend. Our study is important from the species’ conservation perspective and fills a knowledge gap about species distribution in the Balkan Peninsula.

**Abstract:**

Studying current and future geographic distribution is essential for conserving endangered species such as the Boreal Owl and Eurasian Pygmy Owl. The main aim of this study was to determine the potential distribution of both species in the Balkan Peninsula by using spatial distribution models (SDMs) in MaxEnt. We used data from field surveys, the scientific and grey literature, and an online database. We considered the current time and two future periods, 2041–2060 and 2061–2080. For future periods, we included different climate scenarios (SSP 126, 245, 370, and 585) in studying the potential geographic distribution of both species. We identified two types of potential future refugia for species: in situ and ex situ. Our study shows the highly suitable area for the Boreal Owl increased during the 2041–2060 period compared with the current area in all scenarios, except in SSP 585. However, during the 2061–2080 period, the highly suitable areas contracted. For the Eurasian Pygmy Owl, highly suitable areas decreased during 2041–2060, but during the 2061–2080 period, it was larger than the current area. Our study is of importance for conservation and preserving areas of potential distribution and refugia for Boreal and Eurasian Pygmy Owls in the face of climate change.

## 1. Introduction

Biodiversity is a fundamental component of planet life-support systems, and human well-being depends on nature services, such as essential material goods, underpinning functions, and nonmaterial benefits [1,2,3]. However, biodiversity on our planet has been declining at an alarming rate in recent decades. This rate is predicted to be 100 to 1000 times bigger than natural background extinction rates [4,5,6] and is expected to continue at an increasing pace in the forthcoming decades [2,3,4].

Overall, five underlying key drivers cause biodiversity loss and species extinction via many pathways across different physical and temporal scales. These drivers include habitat loss, invasive alien species, overexploitation of natural resources, environmental pollution, and global climate change [7]. Among them, climate change is perceived as the major environmental issue of the 21st century and is anticipated to have vast negative consequences on the planet’s biosphere [8]. The Intergovernmental Panel on Climate Change (IPCC) report predicts that global warming temperatures will likely reach 1.5 °C above preindustrial levels by 2040. Additionally, it is projected to grow by nearly 0.2 °C per decade [8]. Climate change strongly impacts biodiversity at various levels. It shifts species distribution [9,10,11] and migration phenology [12], affects population dynamics [13,14], changes community structure and composition [15], and influences the functioning of entire ecosystems [16,17].

Climate and species geographical distribution are causally related. Predicted global warming is expected to significantly impact the spatial distribution of biota worldwide. For instance, in environments closer to the Equator (i.e., tropics) or that are mountainous, species can be forced to shrink their distributions toward poles or move upslope to higher altitudes to escape warming temperatures and other unsuitable climatic conditions [18,19,20]. These changes in a species’ distribution may jeopardise its persistence by reducing its range or fragmenting the population, leading to population size declines or risk of extinction [21,22,23]. Further, forecasts indicate that the population of habitat-specialised species is decreasing at a notably greater rate than habitat generalists [24,25]. Thus, relatively small biodiversity hotspots could be heavily threatened by climate change [26].

Therefore, over the last 30 years, scientists started studying species distribution modelling, also known as environmental (or ecological) niche modelling (ENM) [27,28,29,30]. This approach is based on mathematical algorithms that use data from presence/absence records and the environmental conditions at occurrence localities [29]. Specifically, modelling is applied but not restricted to predicting the potential geographical distribution [31,32] to recognise habitat suitability and priority areas for conservation [33,34,35], and, more recently, is used to study changes in geographic distribution concerning climate change [24,25,36]. One of the most used species distribution modelling approaches is the machine-learning algorithm MaxEnt (Maximum Entropy), American Museum of Natural History, New York, USA (for details, see [37,38,39,40]). MaxEnt is a favoured and widely applied tool because it demands only presence data, can utilise categorical as well as continuous variables, includes interactions between predictor variables, shows a satisfactory predictive performance, and generally outperforms other SDMs [41,42,43].

The Boreal (*Aegolius funereus*) and Eurasian Pygmy Owls (*Glaucidium passerinum*) are small, forest-dwelling avian predators belonging to the Siberian–Canadian faunal type [44,45]. Consequently, both species are confined to boreal climatic zones and high-mountain regions in the Palearctic (Eurasian Pygmy Owl) and Holarctic (Boreal Owl) realms. In Europe, they are almost sympatric inhabitants of the taiga belt in the northern parts of the continent. At the same time, several small, disjunct populations occur in high-mountain forests in the central and southern parts of the continent [44,45]. Across the European distribution range, both species are highly dependent on old growth (>80 years old), and primarily, coniferous forest stands, choosing dry and dead trees with cavities for breeding and food storage [45]. In Southern Europe, particularly on the Balkan Peninsula, both species prefer higher elevations, north-faced slopes, and medium-to-dense forests with a cold and humid climate [46,47,48]. Therefore, it can be assumed that Boreal and Eurasian Pygmy Owls are stenovalent habitat specialists with a narrow tolerance range and few possibilities of adaptation, which can only survive in the specific, above-mentioned environmental conditions. According to BirdLife International [49,50], there are less than ten thousand mature individuals of Boreal Owls (around 3% of the European population) and no more than six thousand mature individuals of Eurasian Pygmy Owls (about 2% of the European population) in the whole Balkan Peninsula. Knowledge about the spatial distribution range is limited, especially for the Eurasian Pygmy Owl, as well as information on population trends, except for a few countries where it is known that the numbers are decreasing (e.g., Serbia). Additionally, in almost all Balkan countries, both species are assessed as vulnerable or endangered with significant threats, such as forest exploitation and fragmentation, the development of ski resorts, and other human disturbances [51,52]. Further, due to global climate warming, the area comprising Norway spruce (*Picea abies*) in the central Balkan Peninsula, a primary habitat of both species, is expected to decrease, and the range will shift to higher altitudes [53]. Thus, the projected climate change may have a negative impact on the habitat suitability of both species, which may lose remarkable portions of their primary niche. Accordingly, determining the optimal forest habitat patches of both owl species is necessary to understand the role of topographic and climate factors in their potential habitat suitability under present and future climate scenarios.

The aims of this study were: (1) to define the potential current distribution through the development of an SDM and a set of environmental predictor variables; (2) to evaluate which environmental factor(s) influence spatial distribution; (3) to consider the potential impact of climate scenarios on the future distribution; and (4) to recognise potential refugial areas of Boreal and Eurasian Pygmy Owls in the Balkan Peninsula using MaxEnt modelling.

## 2. Materials and Methods

**Figure 1:**
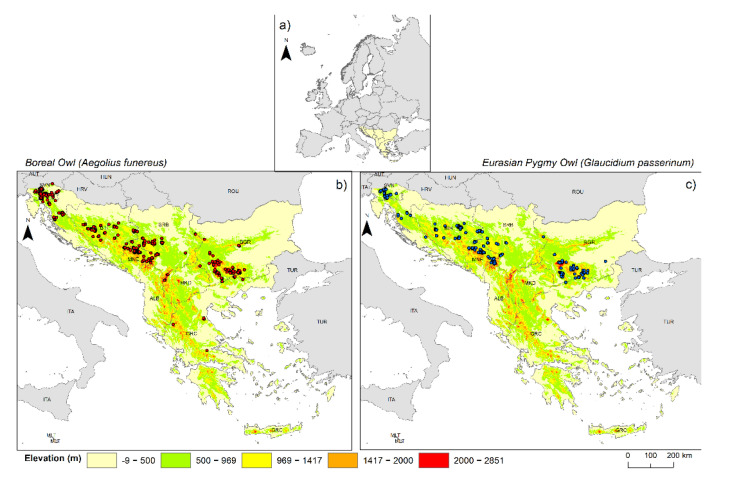
Study area (**a**) at a larger scale. Map of the study area with elevation of the Balkan Peninsula at a smaller scale with points where Boreal Owl (*Aegolius funereus*) (**b**) and Eurasian Pygmy Owl (*Glaucidium passerinum*) were present (**c**).

Our study area, the Balkan Peninsula, extends from Central Europe in the north to the Eastern Mediterranean region in the south, covering approximately 667,000 km^2^, and is surrounded by the Adriatic, Ionian, Aegean, and Black Seas [54] (Figure 1a). Although belted by four seas, the Mediterranean climate is only present on the coast, with mountain ranges preventing warm air from penetrating into other parts of the peninsula [55]. Therefore, the rest of the peninsula is characterised by an alpine climate with strong altitudinal changes in precipitation and temperature, and by the continental climate in the river valleys and lowlands [56]. Due to the variety of climatic conditions, the Balkan Peninsula is one of Europe’s endemism and biodiversity hotspots, as well as a glacial refuge for flora and fauna [57].

Regarding the vegetation cover, at an altitude of 0–700 m, forests comprise the mixed *Fagus* and *Carpinus* communities, with montane forest communities including mostly *Fagus* species [57]. At an altitude of 700–1700 m, the forest community comprises conifers such as *Abies*, *Picea*, and *Pinus*. Above this altitude is alpine vegetation with *Pinus*, *Juniperus*, and *Alnus* [57].

To compile the Boreal and Eurasian Pygmy Owls occurrence data (the geographic coordinates) from across their natural range in the Balkan Peninsula, we used three different sources: (1) an online database [58]; (2) the scientific and “grey” literature; and (3) records from targeted field surveys using GPS devices, which provided most data (>80%) used in this study. The IUCN Red List criteria for the size of the last three generations of a species were followed to provide biologically meaningful data. Therefore, the collected data related to the period from 2002 to 2020 (3 generations = 18 years) for the Boreal Owl and 2008–2020 (3 generations = 12 years) for the Eurasian Pygmy Owl were used. We derived 883 and 584 occurrence points of Boreal Eurasian Pygmy Owls, respectively. It is important to mention that differences in data collecting approaches were not expected to substantially impact the final model results because Maximum Entropy modelling is particularly well suited to handle all kinds of presence-only data [31].

After this initial step, we carefully cross-checked the data, deleted all duplicate records, and discarded data with obvious georeferencing errors. To avoid spatial autocorrelation in occurrence localities, we performed a filtering process of the rest of the occurrence data using the ArcGIS 10.7.1 software [59]. The spatial filter of occurrence localities was limited to 30 arc s between each other (ca. 1 × 1 km resolution at ground level), which is consistent with published data related to the territory density [47,48,49,50,51,52,53,54,55,56,57,58,59,60] and home range size [61,62] of both owl species. Thus, we left only one occurrence record within each grid cell of 1 × 1 km. Additionally, we used the Global Moran’s coefficient for an additional recheck if there was a potential problem with spatial autocorrelation in the occurrence dataset [63]. This index represents the widely used multidimensional and multidirectional statistical tool for measuring spatial autocorrelation in ecological studies [64,65]. We employed the “Spatial Autocorrelation (Global Moran’s I)” tool from ArcGIS (Esri, Redlands, CA, USA) software to calculate the Global Moran index using the nearest neighbour approach. We did not detect autocorrelated data in either the Boreal Owl (Moran’s I = 0.047 *p* = 0.573) or the Eurasian Pygmy Owl (Moran’s I = 0.189 *p* = 0.748). Finally, 439 and 235 precise occurrences of Boreal and Eurasian Pygmy Owls, respectively, were left (Table S1); these data were utilised to create the SDMs and the detailed distribution map (Figure 1b,c).

For modelling the current distribution of the species, we used recent bioclimatic variables, such as elevation, aspect and slope of the mountain, soil classification, snow cover, human footprint index, and land-use type. The sources of the environmental variables are available in Table S2.

Based on the published literature and the authors’ assessment, climate and other predictor variables were selected according to their relevance and importance to owls’ life cycles. For instance, it is generally known that most species, including Boreal and Eurasian Pygmy Owls, inhabit a specific bioclimatic niche which is predominantly regulated by main climatic factors such as temperature and precipitation [66,67]. In this case, both owl species across distributional ranges are associated with cold and humid boreal and high-mountain climate conditions [44,45]. Therefore, we decided to use all 19 different bioclimate variables from Global Climate Data–WorldClim version 2.1 [68] in the initial baseline model (present time, 1950–2000). These variables represent a crucial, ecologically meaningful, and the most applied set of high-resolution global climate layers in SDMs and related ecological modelling techniques [30,69]. In Southeast Europe, particularly in the Balkan Peninsula, Boreal and Eurasian Pygmy Owls, as postglacial relicts, inhabit high mountain areas, preferably above 1000 m a.s.l. Furthermore, they are cold-adapted forest-dwelling species that prefer north-facing, steep, often rocky slopes at higher altitudes covered with old-growth mixed and coniferous forests, usually grown in shallow soil [46,47,48,51,60,70]. In addition to bioclimatic variables, we included the digital elevation model (DEM), slope gradient, aspect, soil type, and hill shade in the initial modelling in this study (Table S2). We did not include other potentially useful layers, such as land use or land cover, due to their high potential variability in time and space, making them unrealistic and irrelevant for modelling distribution patterns in future scenarios. To determine the future distribution of both owl species under contrasting climate scenarios, we used datasets of future climate predictions from Global Climate Data–WorldClim version 2.1 [68]. Four representatives of the Shared Socioeconomic Pathways (SSP126, SSP245, SSP370, and SSP585) ratified by the Intergovernmental Panel on Climate Change (IPCC) [8] were considered in modelling processes related to future climate scenarios and the habitat suitability distribution of both owl species. These SSPs are a part of the Coupled Model Intercomparison Project, Phase 6 (CMIP6) [8]. The four SSPs are defined by the predicted range of radiative forcing values [8]. Predicting suitable species distributions under climate change scenarios involved climate data for the next two periods: 2041–2060 and 2061–2080. All used layers were converted into a spatial resolution of 30 arc seconds (ca. 1 × 1 km resolution at ground level) and trimmed to the Balkan Peninsula shape using ArcGIS software.

We predicted the potential distribution of Boreal and Eurasian Pygmy Owls under different climate change scenarios by applying MaxEnt version 3.4.4. [38]. The MaxEnt program settings undoubtedly significantly influence model performance and prediction power [40]. Although the MaxEnt software can be successfully utilised for SDM purposes with the default settings [31], later studies have convincingly demonstrated that employing automatic features will not generally result in the best prediction model [71,72,73]. Therefore, respecting the calls for prudence and following general recommendations [73,74], we tried to achieve potentially the best combination of feature classes and a regularisation multiplier (β coefficient) to express the best fitting model adequately.

To model habitat suitability for each species (Tables S3 and S4), we developed a comprehensive set of initial models with all 19 BioClim variables plus 5 topographic variables and a β coefficient changing from 0 to 5 in increments of 0.2, resulting in 26 models per owl species. Except for the β coefficient, other MaxEnt parameter settings were kept as the default. Tuning the β coefficient (regularisation multiplier) between 0 and 5 was a standard procedure that aimed to sufficiently reduce overfitting to reasonable levels [39,73]. For each species and each initial model, we used the sample-size-adjusted Akaike information criterion (AICc) [75,76] to determine the most appropriate variable combination and to tune model complexity [32,36]. We retained only the model with the lowest AICc from the initial set for each species, creating a baseline model. Moreover, we calculated the MaxEnt contribution scores for each environmental variable from each baseline model. Predictor variables indicating no remarkable effect on species occurrence with percent contribution scores ≤1% in the baseline model were eliminated. Then, the variable with the highest score was retained and added to the final variable set [77]. All other variables strongly correlated with the retained predictor variable at a pairwise Pearson correlation coefficient of | r | > 0.70 [36,78] were deleted. This process was replicated until all variables were switched to the baseline model set or discarded. Next, we checked the newly established set of the baseline model variables for multicollinearity with the help of a widely used diagnostic quotient: the variance inflation factor (VIF). All variables with a VIF score ≥6 [77,79] were eliminated from further processing, starting with the one with the highest VIF score. This process was repeated until all the remaining variables scored lower than 6. Altogether, 9 predictor variables for the Boreal Owl and 12 for the Eurasian Pygmy Owl were retained as inputs for MaxEnt modelling of Boreal and Eurasian Pygmy Owls in the Balkan Peninsula.

To reduce overfitting and simplify the interpretation [32,34], we only employed linear (L) and quadratic (Q) features and their combination (L + Q) in the finishing stage of the SDMs. This procedure resulted in generating three models per species. As in the previous steps, we retained the model with the lowest AICc to simulate the current and future distributions of the Boreal and Eurasian Pygmy Owls in the Balkan Peninsula. We set the maximum number of iterations to 1000 to allocate the models sufficient time to converge [35]. We applied “maximum training sensitivity plus specificity”, which represents a pretty satisfactory method for threshold selection in the case when only presence data are available [80]. The random test data were 25% of the sample data, and the training data were the remaining 75% of the sample data selected randomly. The habitat suitability curves of each predictor variable were calculated, as were the contributions of each predictor variable using the jack-knife test. All other MaxEnt parameter settings were kept as the default. We used the AUC (area under the ROC curve) to determine which models performed better than others. AUC values range from 0 to 1, with 0 being the lowest performance of the model and 1 being the highest performance of the model.

As metrics for quantifying the similarity among SDMs are important for testing patterns of niche evolution, we calculated the similarity statistic *I* [28]. It ranges from 0 (no overlap) to 1 (identical niche models). The mathematical formula is available in a study by Warren et al. [28].

All statistical tests were performed in RStudio [81].

After choosing the final models, we imported them into ArcGIS and divided habitat suitability into four levels according to the AUC values: unsuitable habitat (0–0.05), poorly suitable habitat (0.05–0.33), moderately suitable habitat (0.33–0.67), and highly suitable habitat (0.67–1). Various studies have different approaches in determining “highly suitable habitat” classification, where some are too strict (0.8–1) [82] and others are more accepting (0.6–1) [83,84,85]. Therefore, we decided to use a classification that would meet the requirements in the middle. According to these levels, we calculated the area of each species distribution under each climatic scenario and for each period, as well as an area of species distribution within each country.

We calculated areas of potential climate refugia for both species by looking at the highly suitable habitats in the current and future species distribution models. We followed the methodology of Brambilla et al. [32], where two types of refugia were identified: type 1 refugia are habitats suitable in both current and future conditions (in situ sites), and type 2 refugia are habitats that are not suitable in current conditions but provide suitable conditions in all future predictions (ex situ sites).

## 3. Results

The current species distribution prediction accuracy for Boreal and Eurasian Pygmy Owls was considered “excellent”, where AUC_mean_ = 0.91 for both species (Table 1 and Table 2). Regarding the environmental variables for both species, bio5 (maximum temperatures of the warmest month) contributed the most to the MaxEnt models (74%). Interestingly, the Boreal Owl was absent in cells with maximum temperatures of the warmest month higher than 31 °C, whereas the Eurasian Pygmy Owl was absent in cells with maximum temperatures of the warmest month higher than 34 °C. The rest of the environmental variables all had less than a 10% contribution to the MaxEnt models. Regarding the current predicted distribution for the Boreal Owl, highly suitable areas cover 261 km^2^, moderately suitable areas cover 447 km^2^, and low suitable areas cover 1992 km^2^ of the entire Balkan Peninsula (Table 3) (see Table S1 per country). For the Eurasian Pygmy Owl, highly suitable areas cover 233 km^2^, moderately suitable areas cover 385 km^2^, and low suitable areas cover 1271 km^2^ of the entire Balkan Peninsula (Table 4) (see Table S2 per country). Both species had the largest areas of highly suitable habitats in Serbia and Bosnia and Herzegovina. Note that the alpine parts of Slovenia are excluded from the analysis since this area does not belong to the Balkan Peninsula.

**Figure 2:**
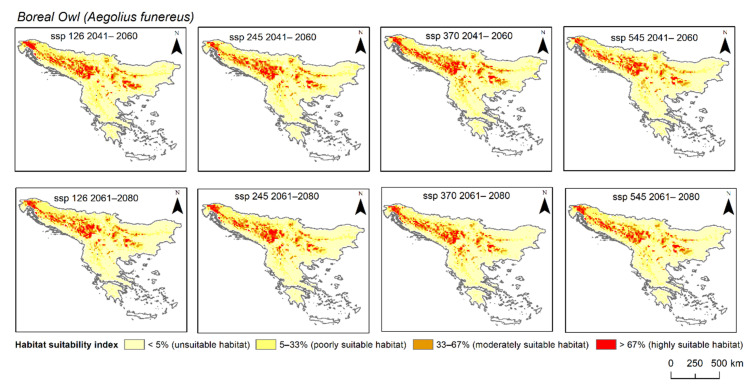
Predicted highly suitable habitat of Boreal Owl (*Aegolius funereus*) under projected future climate scenarios (SSP 126, 245, 370, 585) in two different periods: 2041–2060 and 2061–2080. Colour coding: beige = unsuitable habitat; light yellow = poorly suitable habitat; dark yellow = moderately suitable habitat; red = highly suitable habitat).

**Figure 3:**
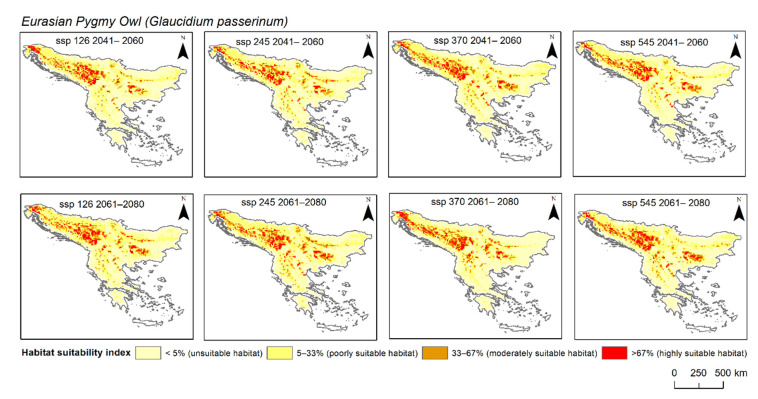
Predicted highly suitable habitat of Eurasian Pygmy Owl (*Glaucidium passerinum*) under projected future climate scenarios (SSP 126, 245, 370, 585) in two different periods: 2041–2060 and 2061–2080. Colour coding: beige = unsuitable habitat; light yellow = poorly suitable habitat; dark yellow = moderately suitable habitat; red = highly suitable habitat).

When looking at the future species distribution models for both species, all four scenarios (SSP 126, 245, 370, and 585) and both periods (2041–2060 and 2061–2080) were considered either “very good” or “excellent” (Table 1). The environmental variable for the Boreal Owl that contributed the most to the model was bio5 (maximum temperatures of the warmest month), with one exception for SSP 370 in 2041–2060, when bio9 contributed the most (69%). However, for the Eurasian Pygmy Owl, apart from bio5, elevation majorly contributed to the MaxEnt models. Regarding the area changes (Figure 2 and Figure 3), specifically for the Boreal Owl, the highly suitable habitat in comparison to the current distribution was only positive, i.e., the area was larger than the current distribution, during the 2041–2060 period for SSP 126, 245, and 370. However, this was not true for SSP 585, where the changes were negative, i.e., the area was smaller than the current distribution. Furthermore, for the entire period of 2061–2080, we found changes to be negative, i.e., smaller than the current distribution. When looking at the area changes for the Eurasian Pygmy Owl, the highly suitable habitat in comparison to the current distribution was only negative during the 2041–2060 period for all scenarios. However, the 2061–2080 models predicted a positive change for all scenarios except for SSP 370.

Despite some changes in spatial distribution between the current and future predictions for both species, an ANOVA did not show statistically significant changes in the DEM (Boreal Owl *p*-value = 0.77, Eurasian Pygmy Owl *p*-value = 0.55).

Results obtained from the similarity statistic *I* showed that the Boreal Owl’s current niche highly overlapped with SSP 126 and 245 in the 2041–2060 period (0.926 and 0.991, respectively). However, when looking at the 2061–2080 period, the current species distribution overlapped highly with all except SSP 585 (0.719) (Table 5). Regarding the Eurasian Pygmy Owl, its current species niche moderately overlapped with all SSPs from the 2041–2060 period, but for the 2061–2080 period, its species niche highly overlapped with all SSPs (Table 5).

**Figure 4:**
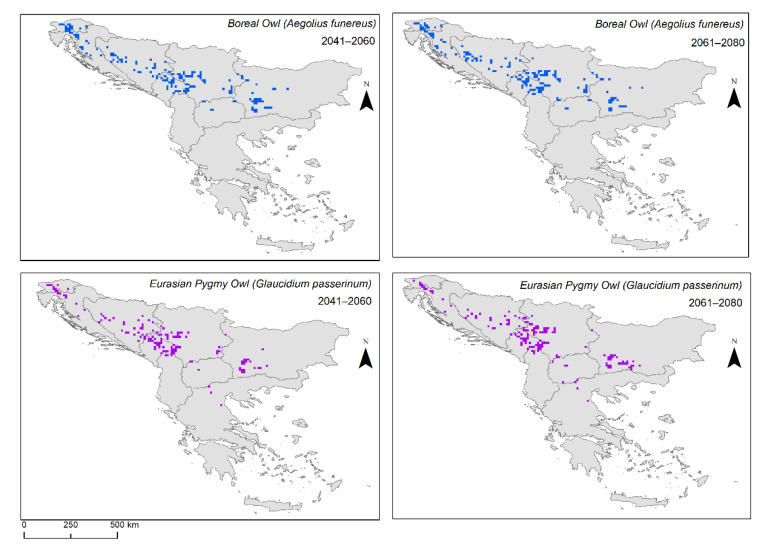
Type 1—in situ refugia for Boreal Owl (*Aegolius funereus*) and for Eurasian Pygmy Owl in the Balkan Peninsula in 2041–2060 and 2061–2080. Colour coding: grey = no refugia; blue = refugia for Boreal Owl; purple = refugia for Eurasian Pygmy Owl.

Our results show that type 1 refugia (in situ) of the Boreal Owl in the periods of 2041–2060 and 2061–2080 reduced its area among the different SSPs (Table 6) (Figure 4). Type 2 refugia (ex situ) followed the same pattern (Table 6). However, for the Eurasian Pygmy Owl, the area of the type 1 refugia in the 2041–2060 period was larger in SSP 126 and 585, and in 2061–2080 it was the largest in SSP 370 and 585 (Table 7). Furthermore, the Eurasian Pygmy Owl had a larger area of type 2 refugia in the 2041–2060 period in SSP 245 and 370, and in the 2061–2080 period, the largest in SSP 126 and 585 (Table 7).

## 4. Discussion

Our results provide the first look at current and future species potential distributions of Boreal and Eurasian Pygmy Owls covering the entire Balkan Peninsula by using MaxEnt modelling. Additionally, our study provides more insight into the environmental and climate variables affecting current and future species distributions. Furthermore, we calculated species area changes and potential refugia at varying temporal scales for these two locally endangered boreal owl species in the face of climate change in the Balkan Peninsula. The outcomes of this study can be utilised to build future conservation strategies, and habitat restoration and management plans for these key, flagship predators of high-mountain habitats in the Balkan Peninsula.

The maximum temperature of the warmest month (bio5) represents the environmental variable that contributes the most to and notably shapes the Boreal Owl’s and Eurasian Pygmy Owl’s habitat suitability and spatial distribution. This is not very surprising since it is well known that high temperatures have a significant influence on boreal species, their distribution, and physiology. When looking at specific temperatures for each species in the current distributions, the Boreal Owl is more sensitive to higher temperatures than the Eurasian Pygmy Owl due to its absence in areas with higher temperatures than 31 °C. Similar results have been reported in a study from the Czech Republic, proving that Boreal owls prefer colder temperatures and higher altitudes [86], and which provides further evidence that species in southern populations, such as in the Balkan Peninsula, are a postglacial relict. The next environmental variable that contributes the most to spatial distribution for the Eurasian Pygmy Owl was elevation. The species prefer higher altitudes, which contradicts the results from the study in the Czech Republic [86]. This is most probably because the Balkan Peninsula has a tree line at higher altitudes than the Czech Republic. Therefore, there is more forest area to inhabit. Altogether, these results suggest a high sensitivity of Boreal and Eurasian Pygmy Owl populations to maximum temperatures of the warmest month. Thus, any significant change in temperatures in the Balkan Peninsula and, probably, through a wider area might affect species potential distributions, as shown in other research for other avian species and geographical areas [87,88].

With global climate change, it is expected that some species will move close to the poles or high elevations [89,90] whereas other species might adapt to these changes [91]. However, in our study, when looking at the changes between current and future species potential distributions, we did not register statistically significant results. We can speculate that this is due to the tree line preventing species from moving to higher altitudes in the future and must consider the limiting factor for both species: higher temperatures at lower altitudes.

All projected distribution models, without exception, show narrow ecological adaptability in both owl species. When looking at the change in future highly suitable areas of the Boreal Owl, a positive change, i.e., the area increases in comparison to the current distribution, is overall present in the 2041–2060 period, except in SPP 585. This was expected, since SSP 585 is considered the worst-case climatic scenario in which CO_2_ emissions rapidly increase until 2080, and then reach the peak at which the trend stabilises [8]. Furthermore, in the period of 2061–2080, only a negative change occurs, meaning the highly suitable area of the species distribution is reduced in comparison to the current species distribution. Regarding future highly suitable area changes of the Eurasian Pygmy Owl, the models showed that for the period of 2041–2060, the area would shrink in its size for each SSP. We can speculate that due to the increased temperatures caused by higher CO_2_ emissions, both species’ highly suitable areas will shrink, since, as it was previously discussed, the species are prone to avoid temperatures above 31 °C and 34 °C. Furthermore, a relatively new study carried out by researchers in the Bulgarian mountains showed that Boreal and Eurasian Pygmy Owls are avoiding inhabiting managed forests and young forests [70]. Even though Serbia and Bosnia and Herzegovina are facing urbanisation of mountainous areas with the development of ski slopes and touristic accommodations that require forest clear cuts [52], these countries still have the largest areas of highly suitable habitats for both species. Unfortunately, the combination of factors such as deforestation and increased temperatures might just be the reason for the loss of highly suitable habitats for these endangered species.

We calculated the type 1 refugia (in situ) of the Boreal Owl, the areas where the species is present currently and where it might be present in the future, under different climate scenarios. These areas are the most important for species conservation since they can enhance populations’ resilience [92]. Our results showed that type 1 refugia would be increasingly contract with the different SSPs toward the worst-case scenario: SSP 585. This result was expected due to the increase in CO_2_ emissions and higher temperatures. Furthermore, the ex situ refugia, type 2, where a species is not present currently but might be in the future, are important for the species’ future redistribution [92]. Our models showed that the Boreal Owl’s potential type 2 refugia would also contract with the different SSPs. However, both types of refugia of the Eurasian Pygmy Owl did not show such a clear trend along the SSPs. Even though our models showed that the future areas of both types of refugia are reducing, these areas are the key habitats for species protection and should be considered targets for conservation. Consequently, declaring these areas as protected areas and managing them accordingly could help support species’ resilience to climate change.

With our study, we filled a knowledge gap regarding both researched species’ current distribution in the Balkan Peninsula. Currently, there are several studies on Boreal and Eurasian Pygmy Owls’ distributions in Serbia [52,93] and Bulgaria [48,51], with unpublished data from Bosnia and Herzegovina, Montenegro, and Croatia. However, little is known about the Boreal and Eurasian Pygmy Owls’ population sizes and distributions in Albania and North Macedonia. Hence, our models of the current distribution of the species are beneficial for species mapping in these areas.

## 5. Conclusions

To safeguard Boreal and Eurasian Pygmy Owls, regular monitoring, habitat preservation, and sustainable management in the Balkan Peninsula are highly required. Special care must be paid to the core areas of both species, i.e., type 1 refugia which can be critical habitat patches for the future survival of both species. In addition, further detailed research is needed to determine how anthropogenic activities affect these two species’ capacity to adapt to changing climatic circumstances.

## Figures and Tables

**Table 1:** Mean AUC values (AUCmean) and standard deviation of the mean AUC values (AUCmeanSD) for the current and future MaxEnt models for Boreal Owl’s distribution under different SSP scenarios.

Periods	SSP	AUC_mean_	AUC_mean_SD
Current		0.91	0.015
2041–2060	126	0.93	0.015
	245	0.89	0.021
	370	0.91	0.023
	585	0.89	0.024
2061–2080	126	0.88	0.023
	245	0.089	0.023
	370	0.86	0.026
	585	0.87	0.025

**Table 2:** Mean AUC values (AUCmean) and standard deviation of the mean AUC values (AUCmeanSD) for the current and future MaxEnt models for Eurasian Pygmy Owl’s distribution under different SSP scenarios.

Periods	SSP	AUC_mean_	AUC_mean_SD
Current		0.91	0.025
2041–2060	126	0.87	0.037
	245	0.88	0.037
	370	0.92	0.016
	585	0.9	0.017
2061–2080	126	0.92	0.017
	245	0.94	0.009
	370	0.9	0.025
	585	0.92	0.019

**Table 3:** Extent of predicted three different categories of suitable habitats (km^2^) for Boreal Owl (*Aegolius funereus*) in current time, and in two periods: 2041–2060 and 2061–2080, in different climate scenarios (SSP 126, 245, 370, 585). Additionally, we calculated changes (%) from current time to future periods.

Years	Scenarios	Predicted Area (km^2^)			Changes in Area (%)		
		Total poorly suitable habitat	Total moderately suitable habitat	Total highly suitable habitat	Total poorly suitable habitat	Total moderately suitable habitat	Total highly suitable habitat
Current	-	1192	447	261			
2041–2060	ssp 126	1355	440	276	13.67	−1.57	5.75
	ssp 245	1270	405	266	6.54	−9.40	1.92
	ssp 370	1266	452	280	6.21	1.12	7.28
	ssp 585	1107	386	222	−7.13	−13.65	−14.94
2061–2080	ssp 126	1255	397	249	5.29	−11.19	−4.60
	ssp 245	1248	399	248	4.70	−10.74	−4.98
	ssp 370	1128	391	247	−5.37	−12.53	−5.36
	ssp 585	1147	386	233	−3.78	−13.65	−10.73

**Table 4:** Extent of predicted three different categories of suitable habitats (km^2^) for Eurasian Pygmy Owl (*Glaucidium passerinum*) in current time, and in two periods: 2041–2060 and 2061–2080, in different climate scenarios (SSP 126, 245, 370, 585). Additionally, we calculated changes (%) from current time to future periods.

Years	Scenarios	Predicted Area (km^2^)			Changes in Area (%)		
		Total poorly suitable habitat	Total moderately suitable habitat	Total highly suitable habitat	Total poorly suitable habitat	Total moderately suitable habitat	Total highly suitable habitat
Current	-	1271	385	233	-	-	-
2041–2060	ssp 126	1279	338	214	0.63	−12.21	−8.15
	ssp 245	1357	359	212	6.77	−6.75	−9.01
	ssp 370	1641	404	206	29.11	4.94	−11.59
	ssp 585	1309	345	215	2.99	−10.39	−7.73
2061–2080	ssp 126	1588	399	238	24.94	3.64	2.15
	ssp 245	1641	454	238	29.11	17.92	2.15
	ssp 370	1451	391	232	14.16	1.56	−0.43
	ssp 585	1457	401	250	14.63	4.16	7.30

**Table 5:** Results of the similarity between species distribution models (SDMs) performed by calculating *I* statistics for Eurasian Pygmy Owl (*Glaucidium passerinum*) and Boreal Owl (*Aegolius funereus*). Comparison of current SDM with each SSP (126, 245, 370, 585) from each period (2041–2060, 2061–2080). *I* statistic ranges from 0–1, 0 being no similarity, and 1 being complete similarity of niche models.

Period	Climatic Scenarios	Eurasian Pygmy Owl	Boreal Owl
		*I* statistic	*I* statistic
2041–2060	Current vs 126	0.857	0.926
	Current vs 245	0.825	0.991
	Current vs 370	0.706	0.882
	Current vs 585	0.872	0.442
2061–2080	Current vs 126	0.991	0.95
	Current vs 245	0.991	0.941
	Current vs 370	0.999	0.932
	Current vs 585	0.986	0.719

**Table 6:** Extent of two types of refugia (km^2^) for Boreal Owl (*Aegolius funereus*). Area of type 1 refugium (in-situ refugium) and type 2 refugium (ex-situ refugium) for each period (2041–2060, 2061–2080) and each SSP (126, 245, 370, 585).

Refugium	Period	SSP	Area (km^2^)
type 1	2041–2060	126	232
		245	218
		370	221
		585	196
type 1	2061–2080	126	214
		245	206
		370	205
		585	207
type 2	2041–2060	126	45
		245	19
		370	14
		585	7
type 2	2061–2080	126	35
		245	23
		370	15
		585	9

**Table 7:** Extent of two types of refugia (km^2^) for Eurasian Pygmy Owl (Glaucidium passerinum). Area of type 1 refugium (in-situ refugium) and type 2 refugium (ex-situ refugium) for each period (2041–2060, 2061–2080) and each SSP (126, 245, 370, 585).

Refugium	Period	SSP	Area (km^2^)
type 1	2040–2061	126	186
		245	168
		370	168
		585	180
type 1	2061–2080	126	184
		245	190
		370	203
		585	198
type 2	2040–2061	126	28
		245	44
		370	38
		585	35
type 2	2061–2080	126	54
		245	48
		370	29
		585	52

## Data Availability

Data are available in the Supplementary Materials, Table S1.

## Supplementary Materials

The following supporting information can be downloaded at: https://www.mdpi.com/article/10.3390/ani12223226/s1, Table S1. Boreal (*Aegolius funereus*) and Eurasian Pygmy Owl (*Glaucidium passerinum*) occurrence coordinates obtained from (1) an online database [5], (2) scientific and grey literature and (3) records from targeted field surveys using GPS devices which includes most data (>80%) used in this study.; Table S2. List of the six predictor variables, used for predicting future distribution of the Boreal and the Eurasian Pygmy Owl, with their sources form where they were obtained.; Table S3. Boreal Owl’s current and future area (km^2^) of highly suitable habitats in each Balkan country.; Table S4. Eurasian Pygmy Owl’s current and future area (km^2^) of highly suitable habitats in each Balkan country.
