# Supplementary material for: Environmental Niche Modelling Predicts a Contraction in the Potential Distribution of Two Boreal Owl Species under Different Climate Scenarios
Source: Animals (Basel). 2022 Nov 21;12(22):3226. doi: 10.3390/ani12223226 (PMC9686532; doi:10.3390/ani12223226)
Supplement: Supplementary file 1 [file animals-12-03226-s001.zip › animals-1981345-supplementary.pdf]

Table S1. Boreal (*Aegolius funereus*) and Eurasian Pygmy Owl (*Glaucidium passerinum*) occurrence coordinates obtained from (1) an online database [5], (2) scientific and grey literature and (3) records from targeted field surveys using GPS devices which includes most data (> 80%) used in this study.

Species	Latitude	Longitude
Aegolius funereus	44.22101	16.83644
Aegolius funereus	43.83642	18.45638
Aegolius funereus	44.18741	18.37719
Aegolius funereus	44.17207	18.28258
Aegolius funereus	44.16781	18.30194
Aegolius funereus	44.17173	18.31666
Aegolius funereus	44.18913	18.28449
Aegolius funereus	44.19173	18.29394
Aegolius funereus	44.19354	18.30965
Aegolius funereus	44.19036	18.31734
Aegolius funereus	44.18468	18.29479
Aegolius funereus	44.1806	18.30181
Aegolius funereus	43.29624	18.73154
Aegolius funereus	43.71486	17.50045
Aegolius funereus	43.70673	17.49699
Aegolius funereus	44.1875	18.37699
Aegolius funereus	44.21441	18.30605
Aegolius funereus	44.18015	18.31191
Aegolius funereus	43.31054	18.72877
Aegolius funereus	43.31575	18.71797
Aegolius funereus	43.32207	18.70735
Aegolius funereus	43.98257	17.73354
Aegolius funereus	43.30484	18.73933
Aegolius funereus	43.30489	18.75393
Aegolius funereus	43.30779	18.76264
Aegolius funereus	43.31418	18.76228
Aegolius funereus	43.9912	17.76828
Aegolius funereus	44.20021	17.02548
Aegolius funereus	44.18539	17.03101
Aegolius funereus	44.16734	17.03486
Aegolius funereus	44.15158	17.05083
Aegolius funereus	44.14357	17.05956
Aegolius funereus	44.13326	17.27665
Aegolius funereus	44.14843	17.2774
Aegolius funereus	44.1565	17.26954
Aegolius funereus	44.3959	16.74346
Aegolius funereus	44.40697	16.7377
Aegolius funereus	44.38549	16.73414
Aegolius funereus	44.38037	16.65128

Aegolius funereus	44.39159	16.60417
Aegolius funereus	44.23511	17.21926
Aegolius funereus	44.21809	17.23399
Aegolius funereus	44.23162	17.23293
Aegolius funereus	44.26911	17.18464
Aegolius funereus	44.26755	17.169
Aegolius funereus	44.08355	17.2502
Aegolius funereus	44.10015	17.25035
Aegolius funereus	44.10033	17.23492
Aegolius funereus	44.08558	17.23467
Aegolius funereus	44.08515	17.21813
Aegolius funereus	44.09935	17.20129
Aegolius funereus	44.09751	17.18739
Aegolius funereus	43.73545	18.27256
Aegolius funereus	43.73073	18.244
Aegolius funereus	43.88861	17.42449
Aegolius funereus	43.86342	17.38073
Aegolius funereus	43.86486	17.39541
Aegolius funereus	43.87854	17.41252
Aegolius funereus	44.27833	17.55893
Aegolius funereus	44.29695	18.01617
Aegolius funereus	43.45417	18.85095
Aegolius funereus	43.8808	17.09754
Aegolius funereus	44.1423	18.43292
Aegolius funereus	44.19973	16.88602
Aegolius funereus	44.30812	18.54866
Aegolius funereus	41.83779	23.38458
Aegolius funereus	41.82355	23.38395
Aegolius funereus	41.82865	23.37865
Aegolius funereus	41.67565	23.55166
Aegolius funereus	41.75154	23.52968
Aegolius funereus	41.84285	23.38904
Aegolius funereus	41.8339	23.4271
Aegolius funereus	41.76696	23.526
Aegolius funereus	41.83665	23.40068
Aegolius funereus	41.78215	23.43343
Aegolius funereus	41.77677	23.44539
Aegolius funereus	41.79613	23.45538
Aegolius funereus	41.79519	23.44296
Aegolius funereus	41.77935	23.45575
Aegolius funereus	41.78968	23.43831
Aegolius funereus	41.77271	23.42934
Aegolius funereus	41.79439	23.48632

Aegolius funereus	41.84519	23.34126
Aegolius funereus	41.84867	23.34162
Aegolius funereus	41.84222	23.34149
Aegolius funereus	41.85329	23.31781
Aegolius funereus	41.87446	24.29696
Aegolius funereus	42.22456	23.42439
Aegolius funereus	42.08228	23.62336
Aegolius funereus	42.25317	23.59392
Aegolius funereus	42.21031	23.39254
Aegolius funereus	42.23214	23.431
Aegolius funereus	42.21678	23.4515
Aegolius funereus	42.23083	23.50794
Aegolius funereus	42.24897	23.29953
Aegolius funereus	42.23058	23.34747
Aegolius funereus	42.24812	23.30988
Aegolius funereus	42.25692	23.30053
Aegolius funereus	41.62184	24.64377
Aegolius funereus	41.61388	24.63161
Aegolius funereus	41.65889	24.25637
Aegolius funereus	41.64953	24.30142
Aegolius funereus	41.76179	24.14196
Aegolius funereus	41.68954	24.13293
Aegolius funereus	41.70488	24.8924
Aegolius funereus	42.22449	23.42443
Aegolius funereus	41.8652	24.30416
Aegolius funereus	41.88789	24.30809
Aegolius funereus	41.82535	24.04466
Aegolius funereus	41.83969	24.01259
Aegolius funereus	41.80041	23.99656
Aegolius funereus	41.81349	23.99137
Aegolius funereus	41.74489	23.96535
Aegolius funereus	41.79915	24.12557
Aegolius funereus	43.45281	22.63336
Aegolius funereus	42.10796	23.32935
Aegolius funereus	42.26626	23.60387
Aegolius funereus	42.17939	23.57872
Aegolius funereus	41.6999	24.38047
Aegolius funereus	41.74461	24.30156
Aegolius funereus	42.65724	24.86389
Aegolius funereus	42.20726	23.12116
Aegolius funereus	42.1947	23.70715
Aegolius funereus	42.68633	24.83084
Aegolius funereus	41.72426	24.58018

Aegolius funereus	41.72204	24.597
Aegolius funereus	41.71304	24.60129
Aegolius funereus	41.70986	24.6096
Aegolius funereus	41.70902	24.62421
Aegolius funereus	41.7029	24.63194
Aegolius funereus	41.69878	24.64123
Aegolius funereus	41.61256	24.62816
Aegolius funereus	41.61438	24.60636
Aegolius funereus	41.62025	24.61404
Aegolius funereus	41.62635	24.60612
Aegolius funereus	41.62338	24.60313
Aegolius funereus	41.61882	24.58387
Aegolius funereus	41.62365	24.56735
Aegolius funereus	41.64173	24.53069
Aegolius funereus	41.6453	24.53644
Aegolius funereus	41.647	24.52601
Aegolius funereus	41.64679	24.55021
Aegolius funereus	43.17814	23.0697
Aegolius funereus	41.51567	23.64118
Aegolius funereus	41.53274	23.64978
Aegolius funereus	43.43959	22.62989
Aegolius funereus	42.17633	22.62285
Aegolius funereus	42.13226	23.33895
Aegolius funereus	41.41124	23.64491
Aegolius funereus	42.47991	23.38377
Aegolius funereus	42.58994	23.23265
Aegolius funereus	42.63239	23.23889
Aegolius funereus	43.43439	22.63936
Aegolius funereus	41.92309	23.37121
Aegolius funereus	41.5497	23.63307
Aegolius funereus	41.81022	24.07755
Aegolius funereus	41.86918	23.90758
Aegolius funereus	44.83064	15.5208
Aegolius funereus	45.40751	14.46691
Aegolius funereus	44.92828	15.4649
Aegolius funereus	44.87701	15.49517
Aegolius funereus	44.94144	15.51684
Aegolius funereus	44.91586	15.5242
Aegolius funereus	45.30255	14.84416
Aegolius funereus	45.33578	14.81525
Aegolius funereus	45.33427	14.79095
Aegolius funereus	45.33869	14.82654
Aegolius funereus	45.28334	14.84237

Aegolius funereus	44.66705	15.06964
Aegolius funereus	44.696	15.05709
Aegolius funereus	44.73737	15.02959
Aegolius funereus	44.76893	15.02845
Aegolius funereus	45.47037	14.73392
Aegolius funereus	44.92759	15.31819
Aegolius funereus	45.42014	15.16966
Aegolius funereus	44.87005	15.60067
Aegolius funereus	44.83624	15.52842
Aegolius funereus	44.74949	15.17443
Aegolius funereus	40.0459	20.7992
Aegolius funereus	41.48021	24.32703
Aegolius funereus	38.81974	22.321
Aegolius funereus	41.33944	24.72885
Aegolius funereus	40.06931	22.40862
Aegolius funereus	41.49965	24.2994
Aegolius funereus	41.48847	24.3226
Aegolius funereus	41.51003	24.35457
Aegolius funereus	40.11827	22.39757
Aegolius funereus	42.03078	20.85402
Aegolius funereus	42.02805	20.86934
Aegolius funereus	42.03124	20.86368
Aegolius funereus	41.82155	20.66639
Aegolius funereus	41.81807	20.64622
Aegolius funereus	43.23931	19.2377
Aegolius funereus	43.04824	19.54495
Aegolius funereus	43.21493	19.25788
Aegolius funereus	43.27316	18.92481
Aegolius funereus	43.03808	19.4449
Aegolius funereus	42.663	20.02061
Aegolius funereus	42.87971	19.71801
Aegolius funereus	42.69229	19.60614
Aegolius funereus	43.08101	19.27795
Aegolius funereus	43.5066	19.18477
Aegolius funereus	43.21904	19.30364
Aegolius funereus	42.76328	20.16427
Aegolius funereus	42.76949	20.14818
Aegolius funereus	43.1959	19.04221
Aegolius funereus	43.2122	19.26784
Aegolius funereus	43.26864	18.9112
Aegolius funereus	43.24681	19.25971
Aegolius funereus	43.52034	19.16038
Aegolius funereus	42.59592	20.03643

Aegolius funereus	42.78293	20.12753
Aegolius funereus	43.23126	19.22834
Aegolius funereus	43.04238	19.4491
Aegolius funereus	43.13495	19.27158
Aegolius funereus	43.07433	19.23351
Aegolius funereus	43.52143	19.14078
Aegolius funereus	43.07958	19.25529
Aegolius funereus	43.07099	19.27782
Aegolius funereus	43.20902	19.31426
Aegolius funereus	43.50157	19.19266
Aegolius funereus	42.60513	20.04309
Aegolius funereus	42.75449	19.97547
Aegolius funereus	42.58062	20.03852
Aegolius funereus	42.70371	19.90698
Aegolius funereus	43.14437	19.3736
Aegolius funereus	43.15294	19.35925
Aegolius funereus	43.14606	19.38381
Aegolius funereus	43.16717	19.33955
Aegolius funereus	43.1774	19.36023
Aegolius funereus	43.18363	19.34999
Aegolius funereus	43.18625	19.3389
Aegolius funereus	43.19186	19.33153
Aegolius funereus	43.27369	19.22769
Aegolius funereus	43.27478	19.20175
Aegolius funereus	43.26323	19.21711
Aegolius funereus	43.25738	19.22547
Aegolius funereus	43.25514	19.23517
Aegolius funereus	43.19505	19.42376
Aegolius funereus	43.19714	19.41393
Aegolius funereus	43.20766	19.39491
Aegolius funereus	43.22694	19.36423
Aegolius funereus	43.23525	19.35989
Aegolius funereus	43.09388	19.2159
Aegolius funereus	43.08645	19.22429
Aegolius funereus	43.06677	19.24745
Aegolius funereus	43.08695	19.25925
Aegolius funereus	43.09595	19.25913
Aegolius funereus	43.1011	19.24957
Aegolius funereus	43.10855	19.24053
Aegolius funereus	43.13366	19.24746
Aegolius funereus	43.06429	19.48049
Aegolius funereus	43.07888	19.47085
Aegolius funereus	43.0801	19.45677

Aegolius funereus	43.04154	19.50567
Aegolius funereus	43.04833	19.49231
Aegolius funereus	43.08316	19.48422
Aegolius funereus	43.08269	19.49603
Aegolius funereus	43.07613	19.5076
Aegolius funereus	43.06841	19.50943
Aegolius funereus	42.7281	19.68858
Aegolius funereus	42.66118	19.83772
Aegolius funereus	43.3369	20.2641
Aegolius funereus	43.3465	20.2669
Aegolius funereus	43.3432	20.2428
Aegolius funereus	43.345	20.2805
Aegolius funereus	43.3392	20.285
Aegolius funereus	43.3321	20.2954
Aegolius funereus	43.2997	20.3167
Aegolius funereus	43.3059	20.3076
Aegolius funereus	43.3274	20.312
Aegolius funereus	43.3171	20.3041
Aegolius funereus	43.4174	20.2719
Aegolius funereus	43.4052	20.2738
Aegolius funereus	43.3242	20.3489
Aegolius funereus	43.3184	20.3871
Aegolius funereus	43.3072	20.3815
Aegolius funereus	43.2969	20.3345
Aegolius funereus	43.3315	20.288
Aegolius funereus	42.7739	21.9551
Aegolius funereus	43.4813	20.8
Aegolius funereus	42.8901	20.4289
Aegolius funereus	42.8612	20.3633
Aegolius funereus	42.8529	20.3598
Aegolius funereus	42.8518	20.3497
Aegolius funereus	43.3078	19.786
Aegolius funereus	43.3138	19.7706
Aegolius funereus	43.308	19.5387
Aegolius funereus	43.3197	19.5319
Aegolius funereus	43.4142	19.8077
Aegolius funereus	43.4078	19.8131
Aegolius funereus	43.4371	19.8251
Aegolius funereus	43.3984	19.8064
Aegolius funereus	43.395	19.7979
Aegolius funereus	43.3928	19.8127
Aegolius funereus	43.3935	19.818
Aegolius funereus	43.3962	19.8461

Aegolius funereus	43.4018	19.8243
Aegolius funereus	43.5988	19.7892
Aegolius funereus	43.5944	19.7821
Aegolius funereus	43.605	19.7691
Aegolius funereus	43.6072	19.7596
Aegolius funereus	43.591	19.8004
Aegolius funereus	43.9364	19.4181
Aegolius funereus	43.9005	19.3371
Aegolius funereus	43.9013	19.3425
Aegolius funereus	43.8955	19.507
Aegolius funereus	43.9154	19.4198
Aegolius funereus	43.9353	19.4036
Aegolius funereus	43.3139	19.7969
Aegolius funereus	43.3313	19.7821
Aegolius funereus	43.3194	20.7628
Aegolius funereus	43.3178	20.7704
Aegolius funereus	43.3189	20.7787
Aegolius funereus	43.3418	20.7803
Aegolius funereus	43.2948	20.7878
Aegolius funereus	43.3373	20.7599
Aegolius funereus	43.309	20.805
Aegolius funereus	43.3224	20.3718
Aegolius funereus	43.3309	20.3696
Aegolius funereus	43.344	20.2845
Aegolius funereus	43.34	20.239
Aegolius funereus	43.8978	19.3247
Aegolius funereus	43.2957	20.7803
Aegolius funereus	43.2817	20.7771
Aegolius funereus	43.2861	20.7829
Aegolius funereus	43.9215	19.3872
Aegolius funereus	43.3344	20.3794
Aegolius funereus	42.9773	20.1163
Aegolius funereus	43.4873	20.7979
Aegolius funereus	43.3816	19.8407
Aegolius funereus	42.8787	20.5005
Aegolius funereus	43.33	20.7712
Aegolius funereus	43.4784	19.8629
Aegolius funereus	43.4167	20.0824
Aegolius funereus	43.4175	20.0606
Aegolius funereus	43.30562	20.78818
Aegolius funereus	43.30497	20.80268
Aegolius funereus	43.32163	20.82266
Aegolius funereus	43.33096	20.74991

Aegolius funereus	43.90499	19.33122
Aegolius funereus	43.91697	19.31749
Aegolius funereus	43.9612	19.31228
Aegolius funereus	43.88391	19.52292
Aegolius funereus	43.32119	20.77983
Aegolius funereus	43.31337	20.35142
Aegolius funereus	43.34713	20.23083
Aegolius funereus	43.29186	20.32342
Aegolius funereus	43.33688	20.27847
Aegolius funereus	43.46126	20.41206
Aegolius funereus	43.33762	20.39075
Aegolius funereus	42.90277	20.44177
Aegolius funereus	42.88914	20.43474
Aegolius funereus	43.30543	19.77421
Aegolius funereus	45.89408	14.51357
Aegolius funereus	45.67716	14.69939
Aegolius funereus	45.74389	14.31681
Aegolius funereus	45.90171	14.45216
Aegolius funereus	45.91664	14.48109
Aegolius funereus	45.91969	14.45062
Aegolius funereus	45.8563	14.40927
Aegolius funereus	45.75261	14.5579
Aegolius funereus	45.80824	14.14564
Aegolius funereus	45.82878	14.11639
Aegolius funereus	45.84123	14.07628
Aegolius funereus	45.87918	14.35638
Aegolius funereus	45.90699	14.47364
Aegolius funereus	45.91671	14.46775
Aegolius funereus	45.89156	14.32541
Aegolius funereus	45.73758	14.64739
Aegolius funereus	45.72841	14.64276
Aegolius funereus	45.8228	14.14563
Aegolius funereus	45.77088	15.31954
Aegolius funereus	45.78553	14.31791
Aegolius funereus	45.56065	14.32836
Aegolius funereus	45.73716	14.69053
Aegolius funereus	45.57458	15.06136
Aegolius funereus	45.67194	14.64701
Aegolius funereus	45.73896	14.30523
Aegolius funereus	45.59868	14.73773
Aegolius funereus	45.67521	14.68838
Aegolius funereus	45.8935	14.46567
Aegolius funereus	45.89071	14.47595

Aegolius funereus	45.9046	14.44193
Aegolius funereus	45.65812	15.05144
Aegolius funereus	45.6855	15.02552
Aegolius funereus	45.83024	14.08027
Aegolius funereus	45.78246	15.3821
Aegolius funereus	45.70396	14.9705
Aegolius funereus	45.75363	14.98381
Aegolius funereus	45.79177	15.38122
Aegolius funereus	45.79805	15.39155
Aegolius funereus	45.73776	15.25349
Aegolius funereus	45.74588	15.26606
Aegolius funereus	45.76845	15.30911
Aegolius funereus	45.86419	14.33501
Aegolius funereus	45.83693	14.3466
Aegolius funereus	45.83006	14.35887
Aegolius funereus	45.61215	14.4953
Aegolius funereus	45.92238	14.32673
Aegolius funereus	45.89613	14.33467
Aegolius funereus	45.81202	14.04246
Aegolius funereus	45.65725	14.95674
Aegolius funereus	46.09609	15.07178
Aegolius funereus	45.86973	14.10646
Aegolius funereus	45.68586	14.44458
Aegolius funereus	45.67513	14.82837
Aegolius funereus	45.59104	14.32025
Aegolius funereus	45.58089	14.42273
Aegolius funereus	45.56941	14.41346
Aegolius funereus	45.87131	14.33829
Aegolius funereus	45.53004	14.44762
Aegolius funereus	45.52908	14.42213
Aegolius funereus	45.53828	14.38651
Aegolius funereus	45.68915	14.70554
Aegolius funereus	45.69011	14.71893
Aegolius funereus	45.7093	14.67018
Aegolius funereus	45.67956	14.64649
Aegolius funereus	45.65581	14.67396
Aegolius funereus	45.69235	14.54584
Aegolius funereus	45.59927	14.39278
Aegolius funereus	45.5914	14.39921
Aegolius funereus	45.57073	14.39226
Aegolius funereus	45.56713	14.37716
Aegolius funereus	45.65029	14.42196
Aegolius funereus	45.72937	14.30789

Aegolius funereus	45.92836	14.4139
Aegolius funereus	45.94894	14.46818
Aegolius funereus	45.88915	14.31231
Aegolius funereus	45.65245	15.06844
Aegolius funereus	45.66145	15.04582
Aegolius funereus	45.72485	14.37342
Aegolius funereus	45.86596	14.39221
Aegolius funereus	45.8725	14.40546
Aegolius funereus	45.86932	14.41834
Aegolius funereus	45.8802	14.41778
Aegolius funereus	45.91951	14.40005
Aegolius funereus	45.94548	14.41091
Aegolius funereus	45.92077	14.42068
Glaucidium passerinum	43.76897	18.24471
Glaucidium passerinum	44.17101	18.38764
Glaucidium passerinum	44.16591	18.28155
Glaucidium passerinum	44.13277	18.45732
Glaucidium passerinum	44.13104	18.4448
Glaucidium passerinum	44.17444	18.40641
Glaucidium passerinum	43.92544	18.65861
Glaucidium passerinum	44.28804	18.61671
Glaucidium passerinum	44.18496	18.29485
Glaucidium passerinum	43.29641	18.73419
Glaucidium passerinum	43.30487	18.7392
Glaucidium passerinum	43.3082	18.73361
Glaucidium passerinum	43.81306	17.43819
Glaucidium passerinum	43.53718	19.09296
Glaucidium passerinum	44.19159	18.30569
Glaucidium passerinum	44.17949	18.39212
Glaucidium passerinum	44.17924	18.30667
Glaucidium passerinum	44.16299	18.4483
Glaucidium passerinum	44.20442	18.31006
Glaucidium passerinum	44.21864	18.31014
Glaucidium passerinum	44.17391	18.37126
Glaucidium passerinum	44.23001	18.30744
Glaucidium passerinum	44.08274	18.37871
Glaucidium passerinum	44.2088	18.27607
Glaucidium passerinum	44.20394	18.29512
Glaucidium passerinum	44.16391	18.41164
Glaucidium passerinum	44.16746	18.42435
Glaucidium passerinum	44.15722	18.42132
Glaucidium passerinum	44.14699	18.42776
Glaucidium passerinum	44.13872	18.38317

Glaucidium passerinum	44.2836	17.56373
Glaucidium passerinum	44.39389	17.02813
Glaucidium passerinum	43.92445	17.04789
Glaucidium passerinum	43.74329	18.04852
Glaucidium passerinum	43.7267	18.25715
Glaucidium passerinum	43.73451	18.22461
Glaucidium passerinum	43.75146	18.24725
Glaucidium passerinum	43.78773	18.26706
Glaucidium passerinum	43.77614	18.29328
Glaucidium passerinum	43.7295	18.26626
Glaucidium passerinum	44.22287	18.2443
Glaucidium passerinum	44.23666	18.20897
Glaucidium passerinum	44.1985	17.01416
Glaucidium passerinum	44.17961	17.01985
Glaucidium passerinum	44.17196	17.02856
Glaucidium passerinum	44.16814	17.01416
Glaucidium passerinum	44.16061	17.03287
Glaucidium passerinum	44.1757	17.0678
Glaucidium passerinum	44.16658	17.05565
Glaucidium passerinum	44.14773	17.06304
Glaucidium passerinum	44.15699	17.0684
Glaucidium passerinum	41.74192	23.54338
Glaucidium passerinum	41.76453	23.52176
Glaucidium passerinum	41.64717	23.41891
Glaucidium passerinum	41.82534	23.36254
Glaucidium passerinum	41.63248	23.49517
Glaucidium passerinum	41.81694	23.42126
Glaucidium passerinum	41.74192	23.54338
Glaucidium passerinum	41.66081	23.41561
Glaucidium passerinum	41.79182	23.44312
Glaucidium passerinum	41.77297	23.46011
Glaucidium passerinum	41.78742	23.48976
Glaucidium passerinum	41.81597	24.08763
Glaucidium passerinum	41.83923	24.01203
Glaucidium passerinum	41.84176	23.96684
Glaucidium passerinum	41.88345	24.31158
Glaucidium passerinum	41.86894	24.2832
Glaucidium passerinum	41.83948	24.30929
Glaucidium passerinum	41.68468	24.84687
Glaucidium passerinum	42.24271	23.62288
Glaucidium passerinum	42.24991	23.6074
Glaucidium passerinum	42.24415	23.59506
Glaucidium passerinum	42.25392	23.59445

Glaucidium passerinum	41.60224	24.62572
Glaucidium passerinum	41.61897	24.6161
Glaucidium passerinum	41.62391	24.60923
Glaucidium passerinum	41.61744	24.60691
Glaucidium passerinum	41.58206	24.56781
Glaucidium passerinum	41.93281	23.98935
Glaucidium passerinum	41.92807	23.98418
Glaucidium passerinum	42.22999	23.42526
Glaucidium passerinum	42.22357	23.44327
Glaucidium passerinum	42.21499	23.39009
Glaucidium passerinum	41.7289	24.59648
Glaucidium passerinum	41.63815	24.54069
Glaucidium passerinum	41.64096	24.53692
Glaucidium passerinum	41.64688	24.54288
Glaucidium passerinum	41.64811	24.53044
Glaucidium passerinum	41.62294	24.56165
Glaucidium passerinum	41.61915	24.58043
Glaucidium passerinum	41.62359	24.59613
Glaucidium passerinum	41.64836	24.12788
Glaucidium passerinum	41.63355	24.66386
Glaucidium passerinum	41.39106	23.59672
Glaucidium passerinum	41.59102	24.80161
Glaucidium passerinum	41.89802	24.06073
Glaucidium passerinum	41.87183	24.29947
Glaucidium passerinum	42.18512	23.56946
Glaucidium passerinum	41.72559	23.43714
Glaucidium passerinum	42.5554	23.2501
Glaucidium passerinum	42	24.15003
Glaucidium passerinum	41.9003	25.8501
Glaucidium passerinum	42.20023	23.61
Glaucidium passerinum	41.9512	24.152
Glaucidium passerinum	42.201	23.601
Glaucidium passerinum	42.16538	23.79158
Glaucidium passerinum	41.84303	24.12909
Glaucidium passerinum	42.19686	23.5761
Glaucidium passerinum	41.97799	23.4707
Glaucidium passerinum	41.73046	24.66396
Glaucidium passerinum	42.26626	23.60387
Glaucidium passerinum	42.19673	23.46298
Glaucidium passerinum	41.61952	24.49041
Glaucidium passerinum	43.90431	16.45022
Glaucidium passerinum	44.94052	15.52013
Glaucidium passerinum	44.91159	15.52884

Glaucidium passerinum	44.82201	15.7191
Glaucidium passerinum	44.80765	15.70525
Glaucidium passerinum	44.72295	15.19378
Glaucidium passerinum	44.82948	15.01454
Glaucidium passerinum	44.8771	15.02599
Glaucidium passerinum	41.48734	24.12795
Glaucidium passerinum	41.51281	24.33224
Glaucidium passerinum	41.50851	24.32492
Glaucidium passerinum	41.49247	24.31052
Glaucidium passerinum	41.50272	24.31026
Glaucidium passerinum	41.49299	24.32619
Glaucidium passerinum	41.48488	24.30864
Glaucidium passerinum	41.49108	24.3042
Glaucidium passerinum	41.47588	24.31448
Glaucidium passerinum	41.55495	24.52848
Glaucidium passerinum	41.49148	24.30253
Glaucidium passerinum	41.42641	24.57421
Glaucidium passerinum	43.07427	19.23363
Glaucidium passerinum	42.56024	19.9998
Glaucidium passerinum	42.70372	19.90698
Glaucidium passerinum	43.52149	19.14223
Glaucidium passerinum	43.08506	19.26122
Glaucidium passerinum	43.04794	19.42974
Glaucidium passerinum	42.58675	20.01552
Glaucidium passerinum	42.59585	20.03641
Glaucidium passerinum	43.16974	19.37944
Glaucidium passerinum	42.57625	20.04038
Glaucidium passerinum	43.06004	19.48154
Glaucidium passerinum	43.1967	19.04584
Glaucidium passerinum	43.5029	19.0725
Glaucidium passerinum	43.09629	19.25732
Glaucidium passerinum	42.70382	19.9147
Glaucidium passerinum	43.05246	19.48838
Glaucidium passerinum	42.75685	19.99781
Glaucidium passerinum	43.27186	18.92816
Glaucidium passerinum	43.09042	19.21976
Glaucidium passerinum	43.27231	18.91677
Glaucidium passerinum	42.61874	19.83671
Glaucidium passerinum	43.28333	19.10614
Glaucidium passerinum	42.79033	20.02299
Glaucidium passerinum	42.66248	20.02055
Glaucidium passerinum	42.72518	19.98545
Glaucidium passerinum	42.81867	19.77059

Glaucidium passerinum	42.8757	19.71728
Glaucidium passerinum	43.0435	19.4545
Glaucidium passerinum	43.30341	18.93219
Glaucidium passerinum	42.57462	19.97887
Glaucidium passerinum	42.76624	19.90968
Glaucidium passerinum	43.0608	19.35949
Glaucidium passerinum	43.29251	18.92668
Glaucidium passerinum	43.04741	19.51784
Glaucidium passerinum	42.76309	19.98987
Glaucidium passerinum	43.19041	19.32841
Glaucidium passerinum	42.58385	20.02474
Glaucidium passerinum	43.04524	19.54205
Glaucidium passerinum	43.03762	19.43946
Glaucidium passerinum	43.03762	19.43914
Glaucidium passerinum	43.0881	19.45425
Glaucidium passerinum	43.07169	19.462
Glaucidium passerinum	43.0678	19.47556
Glaucidium passerinum	43.06653	19.50769
Glaucidium passerinum	43.88387	19.52307
Glaucidium passerinum	43.89771	19.50089
Glaucidium passerinum	43.90578	19.49427
Glaucidium passerinum	43.91319	19.45213
Glaucidium passerinum	43.92623	19.45616
Glaucidium passerinum	43.93731	19.41762
Glaucidium passerinum	43.93326	19.3913
Glaucidium passerinum	43.90465	19.31735
Glaucidium passerinum	43.91522	19.30369
Glaucidium passerinum	43.92186	19.29618
Glaucidium passerinum	43.40495	19.81554
Glaucidium passerinum	42.88785	20.42667
Glaucidium passerinum	42.8601	20.36356
Glaucidium passerinum	42.84897	20.35975
Glaucidium passerinum	42.89536	20.49905
Glaucidium passerinum	43.32547	22.80695
Glaucidium passerinum	43.58536	19.79844
Glaucidium passerinum	43.59899	19.79453
Glaucidium passerinum	43.60558	19.78094
Glaucidium passerinum	43.88275	19.55537
Glaucidium passerinum	43.31872	19.53105
Glaucidium passerinum	43.30844	19.53942
Glaucidium passerinum	43.30549	20.3203
Glaucidium passerinum	43.34821	20.24255
Glaucidium passerinum	43.32827	20.75711

Glaucidium passerinum	43.30239	20.79018
Glaucidium passerinum	42.84932	20.38495
Glaucidium passerinum	43.93778	19.40505
Glaucidium passerinum	43.40159	19.77025
Glaucidium passerinum	43.41635	19.78487
Glaucidium passerinum	45.70587	14.33897
Glaucidium passerinum	45.70521	14.66742
Glaucidium passerinum	45.5658	14.41875
Glaucidium passerinum	45.57138	14.4538
Glaucidium passerinum	45.66136	14.34866
Glaucidium passerinum	45.74547	14.33041
Glaucidium passerinum	45.58383	14.36016
Glaucidium passerinum	45.67583	14.55895
Glaucidium passerinum	45.54498	14.45892
Glaucidium passerinum	45.58269	14.41623
Glaucidium passerinum	45.6415	14.42014
Glaucidium passerinum	45.656	14.47776
Glaucidium passerinum	45.92814	14.4122
Glaucidium passerinum	45.58416	14.49545
Glaucidium passerinum	45.87994	14.241
Glaucidium passerinum	45.74535	14.34948
Glaucidium passerinum	45.71512	15.01345
Glaucidium passerinum	45.82796	14.35089
Glaucidium passerinum	45.8812	14.33518
Glaucidium passerinum	45.90148	14.33235
Glaucidium passerinum	45.90897	14.442
Glaucidium passerinum	45.76016	14.32205
Glaucidium passerinum	45.67518	14.68782
Glaucidium passerinum	45.85436	14.09752
Glaucidium passerinum	45.83251	14.09477
Glaucidium passerinum	45.87023	14.14799
Glaucidium passerinum	45.6366	14.3466
Glaucidium passerinum	45.63661	14.34649

Table S2. List of the six predictor variables, used for predicting future distribution of the Boreal and the Eurasian Pygmy Owl, with their sources form where they were obtained.

Environmental variable	Source	Reference
Bioclimatic variables	Worldclim	https://www.worldclim.org/
Digital Elevation Model (DEM)	Worldclim	https://www.worldclim.org/
Aspect	ArcGIS tool	https://pro.arcgis.com/en/pro-app/2.8/tool-reference/spatial-analyst/aspect.htm
Slope	ArcGIS tool	https://pro.arcgis.com/en/pro-app/2.8/tool-reference/spatial-analyst/slope.htm

Soil type	LUCAS: Land Use and Coverage Area frame Survey	https://ec.europa.eu/eurostat/statistics-explained/index.php?title=Glossary:Land_use_-_cover_area_frame_survey_(LUCAS)
Hill shade	ArcGIS tool	https://pro.arcgis.com/en/pro-app/2.8/tool-reference/spatial-analyst/hillshade.htm
Human footprint index	Wildlife Conservation (WCS)	Last of the Wild Data Version 2, 2005 (LTW-2): Global Human Footprint Dataset (Geographic). Wildlife Conservation (WCS) and Center for International Earth Science Information Network (CIESIN).
Snow cover	NASA Earth Observations (NEO)	https://neo.gsfc.nasa.gov/
Land-use type	Corine Land Cover (CLC)	https://land.copernicus.eu/pan-european/corine-land-cover/clc2018?tab=download

Table S3. Boreal Owl's current and future area (km^2) of highly suitable habitats in each Balkan country.

Current		
	Country	Area (km2)
	Albania	3
	North Macedonia	6
	Bulgaria	47
	Bosnia and Herzegovina	54
	Greece	3
	Montenegro	32
	Slovenia	19
	Croatia	34
	Serbia	63
SSP 126	Period 2041 - 2060	
	Country	Area (km2)
	Albania	3
	North Macedonia	6
	Bulgaria	50
	Bosnia and Herzegovina	55
	Greece	3
	Montenegro	36
	Slovenia	25
	Croatia	29
	Serbia	69
SSP 126	Period 2061 - 2080	
	Country	Area (km2)
	Albania	5
	North Macedonia	5
	Bulgaria	28
	Bosnia and Herzegovina	51
	Greece	0
	Montenegro	38
	Slovenia	20
	Croatia	29

	Serbia	73
SSP 245	Period 2041 - 2060	
	Country	Area (km2)
	Albania	6
	North Macedonia	6
	Bulgaria	37
	Bosnia and Herzegovina	70
	Greece	0
	Montenegro	42
	Slovenia	15
	Croatia	20
	Serbia	70
SSP 245	Period 2061 - 2080	
	Country	Area (km2)
	Albania	5
	North Macedonia	8
	Bulgaria	28
	Bosnia and Herzegovina	47
	Greece	0
	Montenegro	34
	Slovenia	22
	Croatia	26
	Serbia	78
SSP 370	Period 2041 - 2060	
	Country	Area (km2)
	Albania	4
	North Macedonia	6
	Bulgaria	43
	Bosnia and Herzegovina	61
	Greece	0
	Montenegro	38
	Slovenia	19
	Croatia	31
	Serbia	78
SSP 370	Period 2061 - 2080	
	Country	Area (km2)
	Albania	5
	North Macedonia	12
	Bulgaria	34
	Bosnia and Herzegovina	48
	Greece	0
	Montenegro	35
	Slovenia	20
	Croatia	29

	Serbia	64
SSP 585	Period 2041 - 2060	
	Country	Area (km2)
	Albania	1
	North Macedonia	5
	Bulgaria	36
	Bosnia and Herzegovina	53
	Greece	1
	Montenegro	28
	Slovenia	23
	Croatia	26
	Serbia	49
SSP 585	Period 2061 - 2080	
	Country	Area (km^2)
	Albania	3
	North Macedonia	5
	Bulgaria	34
	Bosnia and Herzegovina	53
	Greece	1
	Montenegro	32
	Slovenia	14
	Croatia	23
	Serbia	68

Table S4. Eurasian Pygmy Owl's current and future area (km^2) of highly suitable habitats in each Balkan country.

Current		
	Country	Area (km^2)
	Albania	5
	North Macedonia	11
	Bulgaria	44
	Bosnia and Herzegovina	47
	Greece	6
	Montenegro	33
	Slovenia	10
	Croatia	9
	Serbia	68
SSP 126	Period 2041 - 2060	
	Country	Area (km^2)

	Albania	7
	North Macedonia	3
	Bulgaria	26
	Bosnia and Herzegovina	47
	Greece	5
	Montenegro	35
	Slovenia	18
	Croatia	15
	Serbia	58
SSP 126	Period 2061 - 2080	
	Country	Area (km^2)
	Albania	8
	North Macedonia	10
	Bulgaria	39
	Bosnia and Herzegovina	46
	Greece	6
	Montenegro	42
	Slovenia	16
	Croatia	14
	Serbia	57
SSP 245	Period 2041 - 2060	
	Country	Area (km^2)
	Albania	6
	North Macedonia	5
	Bulgaria	34
	Bosnia and Herzegovina	56
	Greece	5
	Montenegro	35
	Slovenia	12
	Croatia	13
	Serbia	46
SSP 245	Period 2061-2080	

	Country	Area (km^2)
	Albania	6
	North Macedonia	15
	Bulgaria	47
	Bosnia and Herzegovina	42
	Greece	8
	Montenegro	31
	Slovenia	11
	Croatia	15
	Serbia	63
SSP 370	Period 2041 - 2060	
	Country	Area (km^2)
	Albania	7
	North Macedonia	9
	Bulgaria	30
	Bosnia and Herzegovina	52
	Greece	8
	Montenegro	36
	Slovenia	11
	Croatia	14
	Serbia	39
SSP 370	Period 2061-2080	
	Country	Area (km^2)
	Albania	7
	North Macedonia	13
	Bulgaria	45
	Bosnia and Herzegovina	45
	Greece	9
	Montenegro	33
	Slovenia	5
	Croatia	12
	Serbia	63

SSP 585	Period 2041 - 2060	
	Country	Area (km^2)
	Albania	5
	North Macedonia	9
	Bulgaria	31
	Bosnia and Herzegovina	47
	Greece	8
	Montenegro	33
	Slovenia	10
	Croatia	15
	Serbia	57
SSP 585	Period 2061-2080	
	Country	Area (km^2)
	Albania	7
	North Macedonia	14
	Bulgaria	38
	Bosnia and Herzegovina	59
	Greece	11
	Montenegro	36
	Slovenia	11
	Croatia	16
	Serbia	58
